# Decompression in the Management of Large Cystic Lesions Associated With Mixed Dentition Phase: 2 Case Reports

**DOI:** 10.1155/carm/6667997

**Published:** 2025-12-11

**Authors:** Shunan Yan, Jing Lin, Jiayi Chen, Yunze Xuan

**Affiliations:** ^1^ Department of Stomatology, Yanbian University Hospital, Yanji, 133000, Jilin, China, yanbianhospital.com

**Keywords:** ameloblastoma, decompression, dentigerous cyst, large cystic lesion, mixed dentition

## Abstract

**Introduction:**

Large cystic lesions of the jaws in children present unique challenges due to potential damage to developing teeth and facial structures. Traditional aggressive treatments may compromise these structures. Decompression offers a minimally invasive alternative.

**Case Presentation:**

This report describes two pediatric female patients (ages 6 and 9) in the mixed dentition phase presenting with large, asymptomatic mandibular swellings. Diagnostic assessments including clinical examination, panoramic radiography, and histopathology revealed a large cystic lesion initially suspected as odontogenic in origin and later confirmed histopathologically as an ameloblastoma involving the impacted canine and premolar in Case 2. Both lesions caused significant bone destruction and tooth displacement.

**Interventions and Outcomes:**

Both patients underwent surgical decompression under general anesthesia. Case 1 involved decompression via extraction site, while Case 2 involved decompression followed by secondary surgery for definitive treatment after initial biopsy results. Follow‐up over 15–42 months showed significant radiographic bone fill, shrinkage of the cystic cavities, and successful eruption in Case 1 and preservation of the involved teeth in Case 2 of the involved permanent teeth. No nerve damage or recurrence was noted during the follow‐up period.

**Conclusion:**

Within the limitations of this report, decompression appeared to be an effective and conservative management strategy for these large mandibular cystic lesions in the mixed dentition phase, allowing for bone regeneration and preservation of developing permanent teeth.

## 1. Introduction

Cystic lesions of the jaw are frequently encountered in clinical practice [1]. While often asymptomatic, they can expand and cause significant local tissue destruction, tooth displacement, root resorption, malocclusion, or maxillofacial deformation [1]. Traditional management options for large cystic lesions, such as enucleation or resection, can be aggressive, potentially leading to complications like facial deformities, loss of teeth or developing tooth buds, and neurosensory disturbances, particularly undesirable in growing patients [2]. In the treatment of large cystic jaw lesions associated with mixed dentition, preservation of permanent teeth, consideration of subsequent jaw growth, and maintenance of facial contour are paramount [2]. Decompression, a minimally invasive technique, has gained favor as it can reduce the size of the lesion, promote bone formation, and minimize complications [3]. This report presents two cases of large mandibular cystic lesions in pediatric patients during the mixed dentition phase successfully managed with decompression, highlighting its role in minimizing impact on growth and development.

## 2. Case Presentation

### 2.1. Case 1

In December 2018, a systematically healthy 6‐year‐old girl was referred to the clinics of the Department of Stomatology of the Affiliate Hospital of Yanbian University. Her chief complaint was a painless swelling on the posterior mandible, which had been detected 5 days earlier and had not been treated in the meantime. The patient had no relevant personal or family medical history.

#### 2.1.1. Clinical Findings

The left side of the face was more swollen than the right. There was no mandible deviation during mouth opening. Intraorally, the swelling was approximately 2.5 × 2.0 cm in size palpable, firm to touch, with well‐defined boundaries but no paresthesia, or no signs of inflammation on the overlying skin (Figure 1(a)). A panoramic radiograph and CBCT revealed a large, unilocular radiolucency with a well‐defined border, enveloping the crown of the unerupted left mandibular first permanent molar. This tooth was displaced inferiorly near the mandibular border. The developing premolars and second molar bud were also displaced. The distal root of the left mandibular second primary molar appeared resorbed (Figure 1(b)).

Figure 1(a) The intraoral examination showed mandibular vestibular swelling expanded from the gingiva corresponding to the mesial of the left first deciduous molar to the distal buccal side of the left first molar. (b) A patchy low‐density shadow was observed in the right lower first deciduous molar, indicating caries. An oval‐shaped cystic low‐density shadow was also seen in the left mandible.

**Figure:**
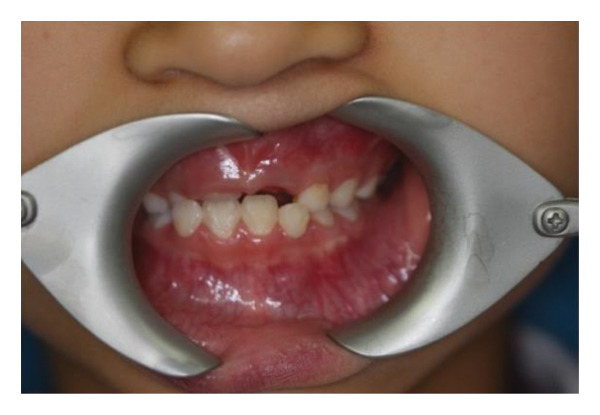
(a)

**Figure:**
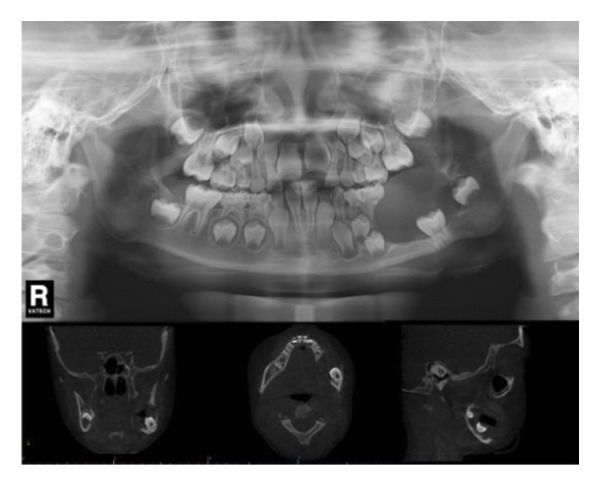
(b)

#### 2.1.2. Diagnostic Assessment

Diagnosis was based on clinical and radiographic findings, suggestive of a dentigerous cyst. Diagnostic challenges included the patient’s young age and the large size of the lesion impacting multiple developing teeth and proximity to the inferior alveolar nerve. A biopsy was planned during surgery for definitive diagnosis. Histopathological examination of the excised cystic lining confirmed the diagnosis of a dentigerous cyst, showing hyalinized fibrous connective tissue with squamous epithelial lining and chronic inflammatory cell infiltration (Figure 2(a)).

Figure 2(a) Hyalinization of a fibrous connective tissue with squamous epithelial hyperplasia and chronic inflammatory cells infiltration. (b) Obvious bone deposition in the cystic cavity and the movement of the teeth. (c) The bone completely healed and the left first permanent molar has erupted. (d) Both the first and second molars were fully erupted.

**Figure:**
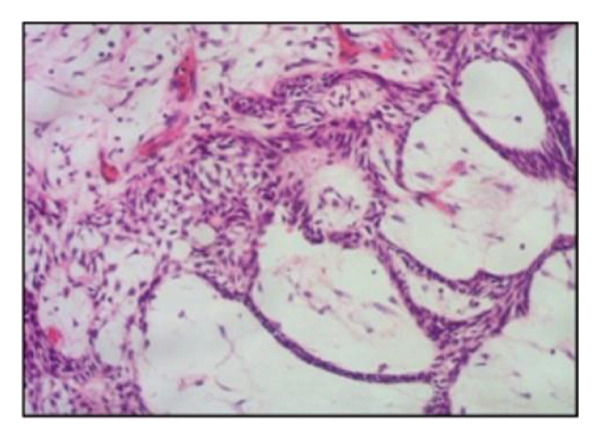
(a)

**Figure:**
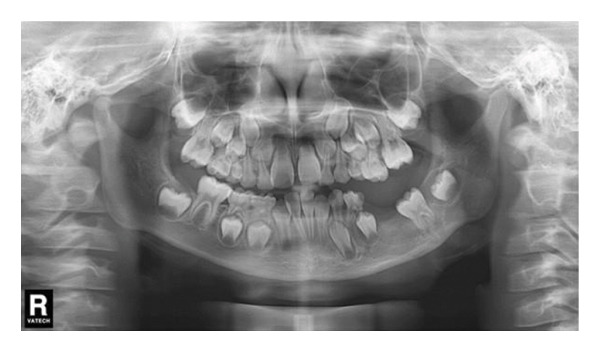
(b)

**Figure:**
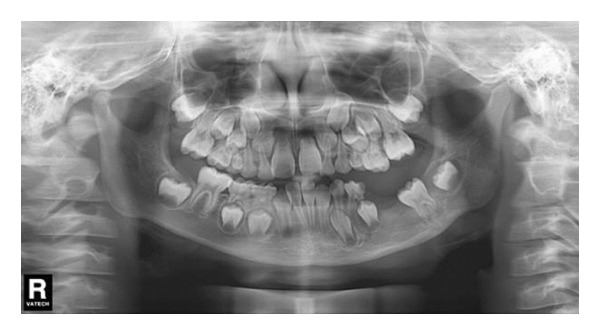
(c)

**Figure:**
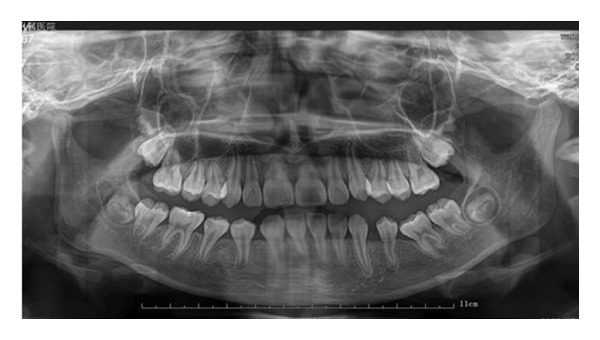
(d)

#### 2.1.3. Therapeutic Intervention

Considering the patient’s young age and poor compliance, surgical treatment under general anesthesia was recommended. The left mandibular second primary molar was extracted to create a surgical window. A mucoperiosteal flap was raised, exposing the cystic wall. A portion of the lining was excised for biopsy. The cystic cavity (approx. 3 cm × 5 cm) contained yellowish fluid. The buccal and lingual cortical plates were resorbed. The cystic wall was noted to be adjacent to the inferior alveolar neurovascular bundle and the crowns of the unerupted first and second permanent molars. The excessively loose left second premolar bud was also extracted during the procedure. The cavity was packed with iodoform gauze, and the incision was partially sutured.

#### 2.1.4. Follow‐Up and Outcomes

To maintain the patency of the decompression window, the patient was required to visit the oral and maxillofacial clinic weekly for the replacement of iodoform gauze and irrigation. After 6 weeks, the iodoform gauze was completely removed. Parents were instructed to irrigate the remaining cavity with at least 10 mL of saline solution after meals and before bedtime until the cavity is completely closed. To maintain the patency of the opening, we fabricated a cyst plug using self‐curing resin and a drainage tube. The parents were concerned about the amount of radiation, so they declined our request to perform a CBCT scan at each follow‐up appointment. Follow‐up panoramic radiographs at 3 months showed evident bone deposition and eruption progress of the involved teeth (Figure 2(b)). At 15 months, complete bone healing was observed radiographically, and the left first permanent molar had erupted into the oral cavity (Figure 2(c)). A follow‐up at 24 months confirmed the full eruption of both the first and second permanent molars (Figure 2(d)). The patient maintained normal sensation in the lower lip and chin throughout the follow‐up period. Adherence was good, confirmed by regular follow‐up attendance and reported irrigation. No adverse events beyond planned extractions occurred. Although the pediatric dentist offered all suitable options to prevent space loss, the patient’s parents refused any type of space maintainer due to financial reasons, which resulted in space loss.

#### 2.1.5. Management of Dressing Changes in Pediatric Patients

Both patients were young children who required long‐term care after surgery. Therefore, we used specific behavior management methods during each dressing change. Before treatment, we informed parents that temporary physical restraint might be necessary if the child could not cooperate due to fear or pain. Both verbal and written consent were obtained, and in accordance with institutional policy, a parent accompanied the child throughout all outpatient procedures to reduce anxiety and improve cooperation.

We always tried nonmedical strategies first. These included distraction, verbal reassurance, the “tell‐show‐do” technique, and positive reinforcement. We suggested sedation if the child could not cooperate, but the parents declined due to safety concerns. When these methods failed, parents helped hold the child briefly, but only during the gauze replacement.

To reduce discomfort, we completed all irrigation and uncomfortable steps before applying any restraint. This helped avoid risks like coughing or choking. We performed each procedure gently and quickly while constantly monitoring the child. This brief and minimal use of restraint followed all hospital protocols and ethical guidelines.

### 2.2. Case 2

In July 2019, a 9‐year‐old systematically healthy girl was referred to request treatment because a large cystic lesion was detected on ordinary radiographic evaluation in another clinic 4 days ago.

#### 2.2.1. Clinical Findings

Extraoral examination revealed slight facial asymmetry on the left side. Intraoral examination showed vestibular swelling extending from the left premolar region to the right first deciduous molar area (Figure 3(a)). A panoramic radiograph and a mandible CT showed a large, unilocular radiolucency with well‐defined borders, causing significant bone destruction extending to the inferior border of the mandible. The impacted permanent left mandibular canine and first premolar were displaced; the canine was near the inferior border, and the first premolar crown was tilted mesially (Figure 3(b)).

Figure 3(a) The mandibular vestibular swelling expanded from the left premolar to right first deciduous molar. (b) A radiolucent unilocular lesion with well‐defined boundary caused destruction of the bone and displacement of permanent canine and left mandibular first premolar bud.

**Figure:**
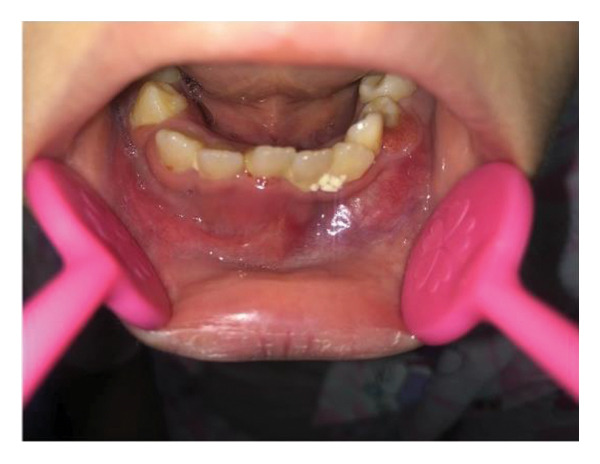
(a)

**Figure:**
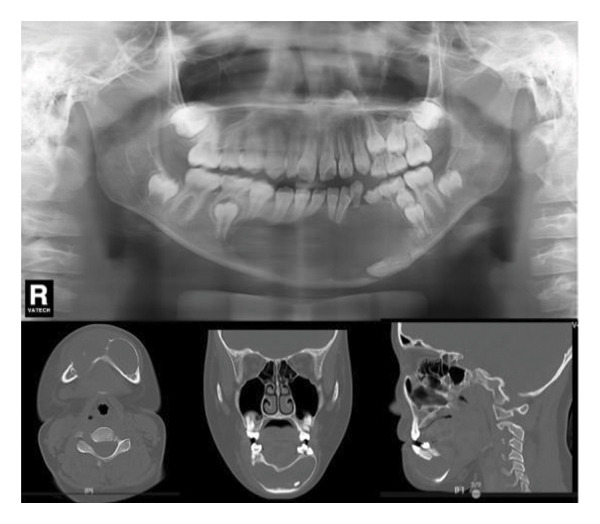
(b)

#### 2.2.2. Diagnostic Assessment

Based on clinical and radiographic findings, a large odontogenic cyst or tumor was suspected. The size, location, and impact on teeth posed diagnostic challenges. An intraoperative biopsy during the initial decompression surgery revealed features of a plexiform‐type ameloblastoma, with tumor cells arranged in anastomosing cords and network‐like patterns (Figure 4(a)). A secondary surgery in January 2020 provided further tissue samples, confirming the plexiform variant of ameloblastoma, showing interconnected basaloid cords with areas of stromal cystic degeneration and focal granulocyte infiltration (Figure 4(b)).

Figure 4(a) Plexiform‐type ameloblastoma: histological examination revealed tumor cells forming anastomosing cords and network‐like structures composed of basaloid epithelial cells. (b) Plexiform type ameloblastoma: histology showed basaloid tumor cells arranged in interconnected cords and plexiform structures. The tumor stroma exhibited focal cystic degeneration and local infiltration of granular inflammatory cells.

**Figure:**
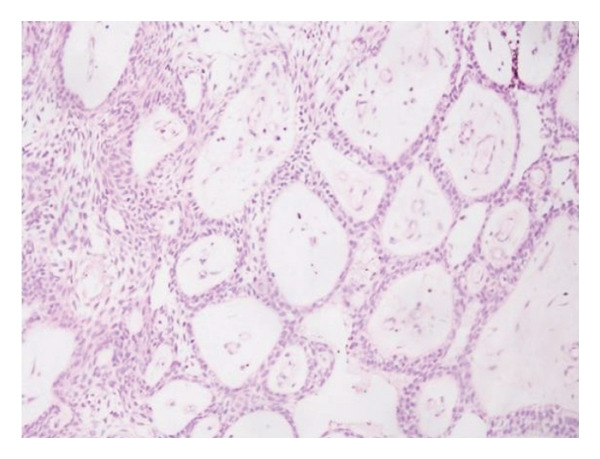
(a)

**Figure:**
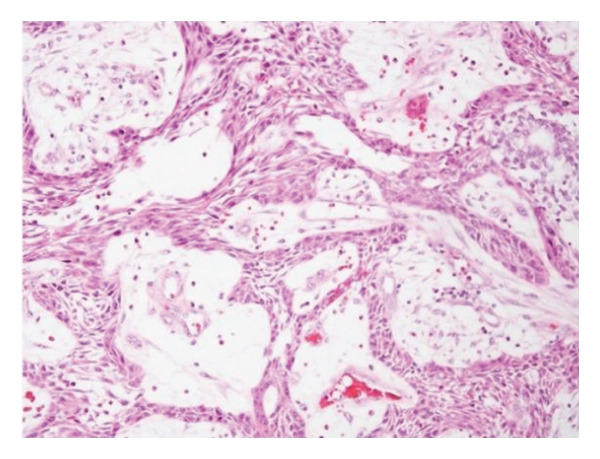
(b)

#### 2.2.3. Therapeutic Intervention

Decompression was performed under general anesthesia. The left primary canine and first primary molar were extracted to create a window. A mucoperiosteal flap was raised. The impacted left permanent canine was also extracted due to its position and involvement. A portion of the cystic wall was excised for biopsy. The cavity was packed with iodoform gauze and partially sutured. The initial biopsy result was suspicion of ameloblastoma. As with Case 1, the patient’s iodoform gauze dressings were changed weekly. Patency was maintained through repeated gauze changes and subsequent irrigation. After 6 weeks, we replaced the iodoform gauze with the drainage plug. Despite our thorough explanation of the impact of CBCT radiation exposure on patient health, the parents still refused to allow a CBCT scan at each follow‐up appointment. In the panoramic radiograph taken 1 month after the first surgery, minimal new bone formation was observed (Figure 5(a)). In the panoramic image taken 3 months later, it can be clearly seen that new bone formation has occurred (Figure 5(b)). After 5 months, radiographic findings showed lingual tooth displacement, and new bone formation remained unsatisfactory. The secondary surgery was performed in January 2020 (Figure 5(c)). We used a round bur to remove the residual cavity instead of a segmental bone resection. Given the patient’s age and the predominantly labial extent of the lesion, the remaining impacted premolars were preserved. The labial bone was removed for access, and the lesion was bluntly dissected for definitive biopsy.

Figure 5(a) Limited new bone formation was observed. (b) New bone reformed. (c) The lingual tooth displaced, but the formation of new bone was not satisfactory.

**Figure:**
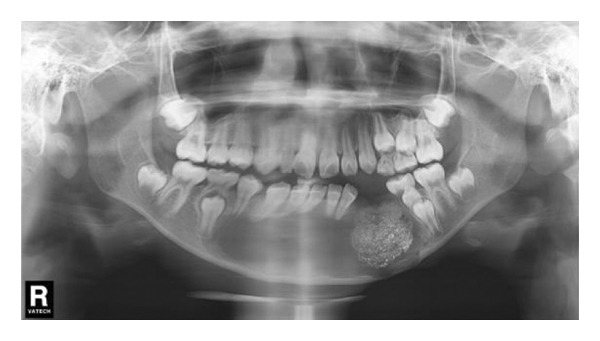
(a)

**Figure:**
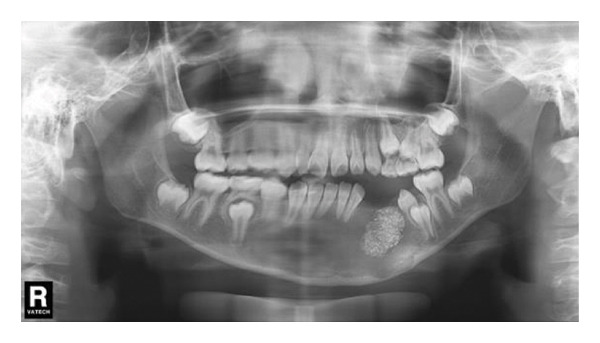
(b)

**Figure:**
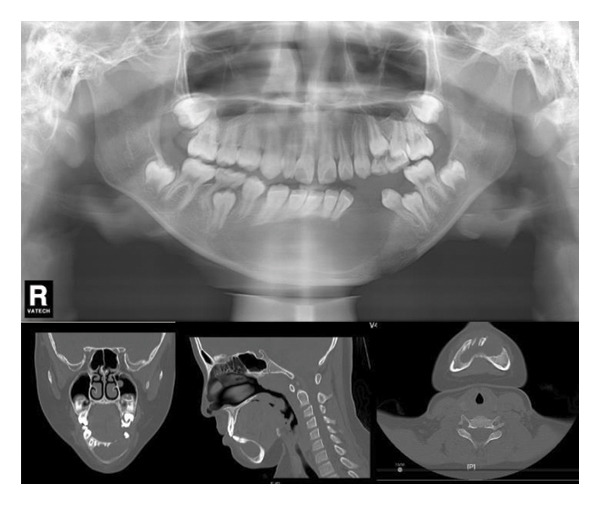
(c)

#### 2.2.4. Follow‐Up and Outcomes

A panoramic radiograph 2 months after the secondary surgery showed bone deposition (Figure 6(a)). Five months later, the left first and second premolars erupted, albeit with mesial inclination, and continued root development of adjacent incisors was noted (Figure 6(b)). In the panoramic image taken 1 year later, the patient’s bone formation was satisfactory (Figure 6(c)). At the 5‐year follow‐up visit, the patient’s panoramic radiograph showed no sign of recurrence (Figure 6(d)). The patient’s family plans to restore the missing teeth with dental implants after the patient becomes an adult. The patient maintained normal facial sensation. Adherence was good through regular follow‐ups.

Figure 6(a) The cavity shrunk and bone formed in it. (b) Five months later, the left first and second premolars were erupted with mesial inclination and the impacted root tips of the left central incisors and lateral incisor continued to develop. (c) One year later, the patient’s bone formation was satisfactory. (d) The new panoramic radiograph revealed complete bone repair and did not relapse.

**Figure:**
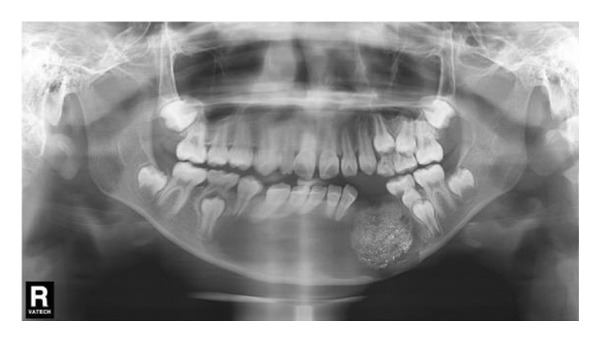
(a)

**Figure:**
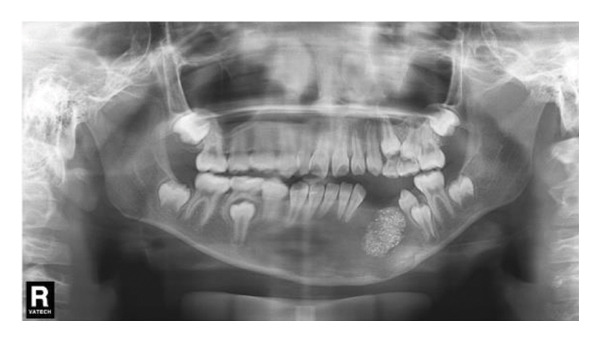
(b)

**Figure:**
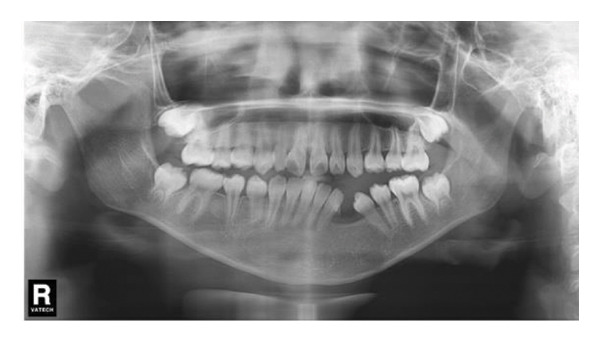
(c)

**Figure:**
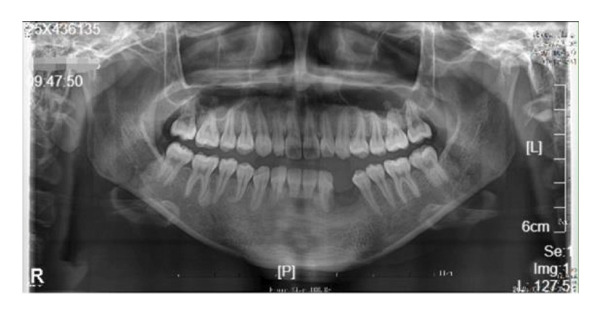
(d)

## 3. Case Summary Table

The key characteristics and outcomes of both cases are summarized in Table 1.

**Table 1:** Summary of two pediatric cases, including age, diagnosis, treatment protocol, follow‐up duration, and outcomes.

Case	Age/sex	Diagnosis	Treatment steps	Follow‐up duration	Outcomes
1	6/F	Dentigerous cyst	Decompression with weekly iodoform gauze replacement ⟶ Gauze removal after 6 weeks ⟶ Replaced the iodoform gauze with the drainage plug⟶ Home saline irrigation	24 months	Complete bone regeneration, eruption of permanent molars, no recurrence
2	9/F	Plexiform ameloblastoma	Decompression with weekly iodoform gauze replacement ⟶ Gauze removal after 6 weeks ⟶Replaced the iodoform gauze with the drainage plug ⟶ Home saline irrigation ⟶ Secondary surgery after 1 year	66 months	Substantial bone regeneration, preservation of premolars, no recurrence

## 4. Discussion

Cystic lesions are the most prevalent jaw diseases. The dentigerous cyst is the second most common cyst of the jaws after the radicular cyst, accounting for 16.6% of all jaw cysts [4], but its exact etiology is still unclear. It often encloses the crown of an impacted tooth and is attached to the cementoenamel junction of the impacted tooth [5]. Ameloblastoma is the most common benign odontogenic tumor, accounting for approximately 1% of all jaw tumors and 11% of all odontogenic tumors [6]. It is a type of aggressive tumor with a tendency to recurrence [6]. The fundamental principle of decompression is the reduction of hydrostatic pressure within the lesion, which removes the stimulus for bone resorption and allows the body’s natural osteogenic potential to take over [8]. This is particularly effective in children and adolescents due to their higher metabolic activity and bone remodeling capacity [8]. As demonstrated in Case 1, this physiological response can be sufficient to achieve complete resolution of a large dentigerous cyst, facilitating the natural eruption of previously impacted teeth without the need for more aggressive surgery.

## 5. Advantages of Decompression

The primary strength of the approach in these cases lies in the use of decompression as a conservative initial strategy. This aligns with literature suggesting decompression (or marsupialization, a related technique) as a favorable option for large cysts in children [8, 9].

It reduces intracystic pressure, allowing for gradual shrinkage of the lesion and stimulating bone regeneration, likely due to the enhanced osteogenic potential in younger individuals [10]. This conservative approach minimizes the risk of damage to vital structures like the inferior alveolar nerve and, crucially in the mixed dentition phase, preserves developing permanent teeth, facilitating their potential eruption [8]. The two cases presented offer a compelling comparison. In Case 1, decompression served as a definitive treatment. It fully resolved the lesion while preserving vital structures, achieving the ideal therapeutic goal with minimal intervention. In contrast, Case 2 illustrates the role of decompression as a crucial preparatory stage for a subsequent, more conservative surgery. Ameloblastoma, being an aggressive benign tumor with a high recurrence rate, typically warrants more aggressive treatment than a cyst. However, a primary radical resection in a 9‐year‐old would have been morbid. By first using decompression, we had increased the thickness of the jawbone in areas such as the lower border of the mandible, thereby laying a foundation for subsequent treatment. This “decompression‐then‐curettage” approach, while conservative, proved effective, preserving the continuity of the mandible and adjacent teeth, with no evidence of recurrence after 5 years. This staged approach is a judicious balance between oncologic control and the preservation of function and form in a growing child.

## 6. Advantages Over Traditional Enucleation in the Pediatric Population

For large lesions in mixed dentition, primary enucleation carries substantial risks. These include iatrogenic fracture of the weakened mandible, damage to the inferior alveolar nerve, and, critically, the unintentional removal of or damage to developing permanent tooth buds. Decompression effectively mitigates these risks [11]. Although it requires longer treatment and high patient compliance, the benefits include a significant reduction in surgical morbidity and preservation of the child’s developmental potential.

## 7. Disadvantages of Enucleation

Traditional enucleation, while offering rapid removal, carries higher risks in such large lesions, including nerve injury, jaw fracture, and damage to tooth buds [2]. Decompression avoids these immediate risks, although it requires longer follow‐up periods and excellent patient/parent cooperation for irrigation and monitoring [8].

## 8. Limitations of This Report

1. Limitations of this report include the small number of cases (two) and the lack of quantitative assessment of bone fill. Future studies may incorporate CBCT or software‐based volumetric analysis. Furthermore, while the follow‐up period was substantial (up to 66 months), longer‐term monitoring, especially for the ameloblastoma case, is always warranted due to recurrence potential [7]. Although one case is not enough to evaluate the clinical effect of decompression combined with curettage for treating large mandibular ameloblastoma in children, we will collect more samples for further research in the future.

## 9. Summary

The rationale for choosing decompression was driven by the patients’ young age, the presence of critical developing teeth within or adjacent to the lesions, and the desire to avoid the morbidity associated with more aggressive primary procedures. The successful outcomes in both cases, despite the different pathologies, support this rationale.

## 10. Conclusion

Within the limitations of Case 1, decompression appears to be a conservative and effective treatment modality for managing large mandibular cystic lesions in pediatric patients during the mixed dentition phase. In Case 2, the treatment of a child with ameloblastoma using decompression combined with curettage also achieved satisfactory results during long‐term follow‐up. This approach facilitated substantial bone regeneration, allowed preservation and eruption of developing permanent teeth, and minimized immediate surgical morbidity. Successful outcomes depend on careful case selection, appropriate surgical technique, parental compliance with irrigation protocols, and diligent long‐term follow‐up. Further studies with larger case series and quantitative imaging assessments are warranted to confirm these findings and refine treatment protocols.

## Ethics Statement

Written informed consent was obtained from the parents of both patients for the surgical procedures and for the publication of these case reports. Institutional ethical approval was obtained from the Ethics Committee of Yanbian University Hospital (Approval No. 2024366).

## Conflicts of Interest

The authors declare no conflicts of interest.

## Author Contributions

Shunan Yan and Jing Lin contributed equally to this work and should be considered co‐first authors.

## Funding

No funding was received for this manuscript.

## Data Availability

The data that support the findings of this study are available on request from the corresponding author. The data are not publicly available due to privacy or ethical restrictions.
